# Fine-resolution profile-scale data to depict the impact of tillage treatment and machine traffic on agricultural soil structure and hydrologic properties

**DOI:** 10.1016/j.dib.2023.109759

**Published:** 2023-11-04

**Authors:** Alice Alonso, Manuel Froidevaux, Mathieu Javaux, Eric Laloy, Samuel Mattern, Christian Roisin, Marnik Vanclooster, Charles Bielders

**Affiliations:** a Earth and Life Institute Environnemental Sciences, Université catholique de Louvain, Place Croix du Sud 1, 1348 Louvain-la-Neuve, Belgium; b Belgian Nuclear Research Centre, Institute for Environment, Health and Safety, Boeretang 200, 2400 Mol, Belgium; c Public Service of Wallonia, Belgium; d Walloon Agricultural Research Center, rue de Liroux 9, 5030 Gembloux, Belgium

**Keywords:** Agricultural soil tillage, Soil traffic, Soil physical quality, Soil hydraulic properties, Soil heterogeneity, Penetrometer

## Abstract

This data article provides high spatial resolution (1 cm) datasets and related figures of the penetrometer resistance (PR) and soil bulk density (BD) data of nine agricultural 50 × 160 cm soil profiles exposed to three tillage treatments and including a wheel track. Soil treatments are moldboard plowing (MP), deep loosening (DL), and minimum tillage (MT). It also provides bulk density data, soil moisture content at various suctions and the parameters of van Genuchten's model for 27 soil cores, and saturated hydraulic conductivity (Ks) of 49 soil cores. Both sample sets were sampled to cover the profile heterogeneity in two agricultural plots subjected to moldboard plowing and minimum tillage. Examples of reuse potential include (i) the use of these spatially explicit data in studies seeking to understand better and integrate the effect of treatment and machine traffic-induced soil structure in soil hydraulic and soil physical quality, and (ii) the development of pedotransfer functions with data incorporating the soil structural heterogeneity. This Data in Brief article complements the companion paper by Alonso et al. (2021) “A hybrid method for characterizing tillage-induced soil physical quality at the profile scale with fine spatial detail” in *Soil and Tillage Research*[1].

Specifications Table

**Table:** 

Subject	Soil Science
Specific subject area	Effect of treatment and machine traffic on soil structure; the relationship between soil structure and soil hydraulic properties.
Type of data	Dataset Graph Figure
How data were acquired	- Fully-automated penetrometer as described in [2] - Soil core sampling and laboratory analysis (sandbox apparatus and low and high-pressure chambers (® Eijkelkamp) and constant head permeameter - Inversion of van Genuchten's water retention curve model [3]
Data format	Raw Analyzed Filtered
Description of data collection	- Penetrometer resistance measurements covered 160 cm (*x*-axis, perpendicular to the tillage direction) × 80 cm (*y*-axis, in the tillage direction). Measurements were taken along a 5 cm resolution grid in the 160 × 80 cm horizontal plane, between 0 and 50 cm depth, with 1 cm increments. - The soil cores were sampled to represent the soil structure heterogeneity based on the methodology developed by Manichon (1987) [4].
Data source location	Institution: Université catholique de Louvain, Earth and Life Institute City/Town/Region: Brabant Wallon (Gentinnes) Country: Belgium Latitude and longitude (and GPS coordinates, if possible) for collected samples/data: 50.5880°N - 4.5948°E
Data accessibility	Figures and graphs are with the article. Repository name: Mendeley Data Data identification number: doi:10.17632/sk2bghhpn8.2 URL: https://data.mendeley.com/datasets/sk2bghhpn8/2
Related research article	Alonso, A., Froideveaux, M., Javaux, M., Laloy, E., Mattern, S., Vanclooster, M., & Bielders, C. (2021). A hybrid method for characterizing soil physical quality at the profile scale with fine spatial details. Soil and Tillage Research, 216, 105,236. https://doi.org/10.1016/j.still.2021.105236

## 1 Value of the Data

• Soil structural features of physical origin are instrumental in the regulation of soil hydrologic response, both quantitatively and qualitatively. However, they are often poorly considered in local to global scale models or in the training of pedo-transfer functions [5]. The provided data enable a more comprehensive assessment and understanding of how agricultural soil treatment and machine traffic influence soil structure, subsequently affecting soil physical quality and related hydrologic behaviors.
• Thus, the data can be useful for soil physic researchers interested in studying the effect of soil structure on hydrologic properties. Such insight is also of interest to the Earth System Model community. The data can also be of interest to soil physics researchers interested in pedo-transfer functions and agricultural researchers studying the impact of soil treatment and compaction on soil physical quality.
• The data might hence be used for a variety of purposes, including:
  (i) A qualitative analysis of the effect of tillage and vehicle traffic through the visualization of PR and BD profiles;
  (ii) The parameterization of soil hydraulic models that take into account the effect of soil tillage and vehicle traffic on the soil structure;
  (iii) A meta-analysis by pooling the data with comparable datasets to compare the relationships between BD and Ks or the parameters of van Genuchten's model [3]. Such a meta-analysis could be conducted to investigate the effect of soil texture on such relationships.
  (iv) The training of pedo-transfer functions using data that capture the soil profile heterogeneity.

## 2 Data Description

**Dataset *SoilSampleParameters_RC.csv/.mat* -** Matrix of 27 rows by 20 columns providing, for each soil sample, the (x,z) sampling location in the soil profile, the bulk density, soil moisture content at various suctions *h* = 1, 10, 31, 63, 100, 310, 1000, 3100, 10,000 and 13,100 cm, the parameters of the vanGenuchten'ss model obtained by inversion ($\theta_{res},\;\theta_{sat},$ α,n,and m) and a quality flag. Metadata is provided with the data files.

**Dataset *SoilSampleParameters_Ks.csv/.mat –*** Matrix of 29 rows by six columns providing, for each soil sample, the (x,z) sampling location in the soil profile, the bulk density, and the saturated hydraulic conductivity.

**Dataset *PR3DA1MP/DL/MT1-3.csv/PR3D.mat* –** 9 matrices of 17,301 rows by four columns containing 3D penetration resistance data for the three repetitions of the three types of treatment (MP, DL, MT). Metadata is provided with the data files.

**Dataset *PR2DA1MP/DL/MT1-3.csv/PR2D.mat*:** 9 matrices of 8211 rows by three columns containing 2D penetration resistance data for the three repetitions of the three types of treatment (MP, DL, MT). Metadata is provided with the data files.

**Dataset *BD2DA1MP/DL/MT1-3.csv/BD2D.mat*:** 9 matrices of 8211 rows by three columns containing 2D bulk density data for the three repetitions of the three types of treatment (MP, DL, MT). Metadata is provided with the data files.

**Fig. 1:**
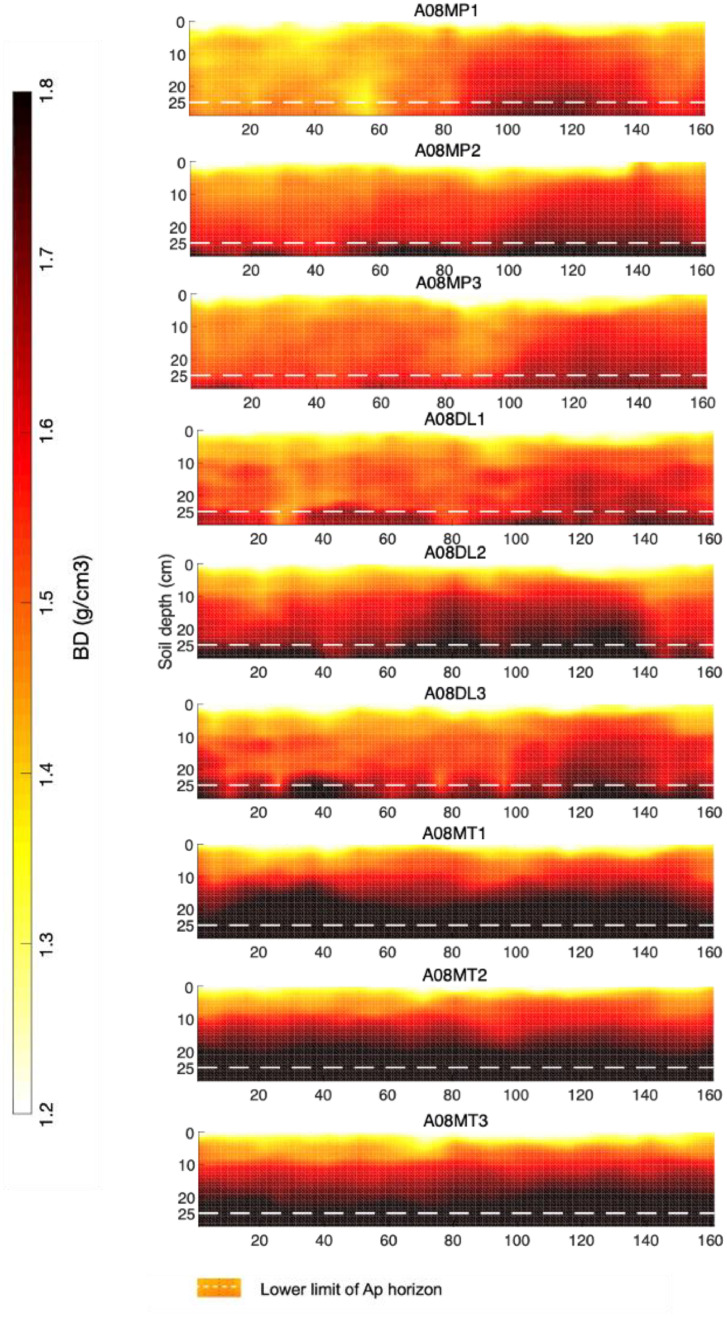
Bulk density 2D data profiles for the three repetitions of the three types of treatment (moldboard plowing (MP), deep loosening (DL), and minimum tillage (MT).

Fig. 1: Visualization of the bulk density 2D data profiles for the three repetitions of the three types of treatment (MP, DL, MT).

**Fig. 2:**
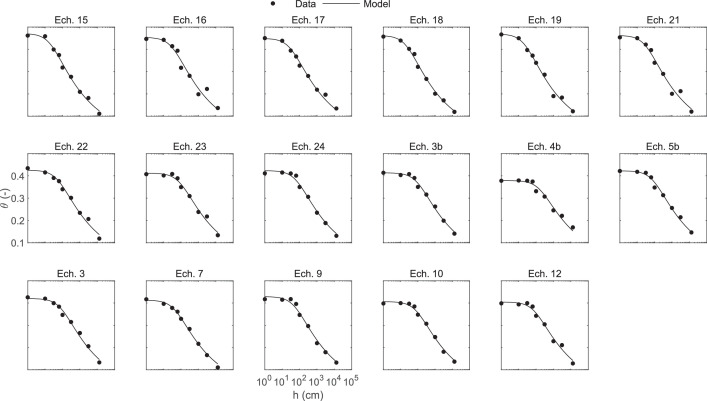
Measured volumetric soil moisture content as a function of suction and the moisture retention curve obtained with the van Genuchten soil moisture retention model.

Fig. 2: Figure of the sample-measured soil moisture retention as a function of suction and the moisture retention curve obtained with the van Genuchten soil moisture retention model.

## 3 Experimental Design, Materials, and Methods

### 3.1 Resistance to penetration

The measurements were conducted on an agricultural field of loamy texture (∼ 18 % clay, 75 % loam, 7 % sand). The field was chosen because it represents the dominant soil types of the Belgian loess belt used for intensive industrial cropping and because of ongoing experiments by the Walloon Agricultural Research Center (CRAW) comparing different tillage techniques. The field included plots that underwent different soil tillage treatments: moldboard plowing (MP) to a depth of 27 cm, which corresponds to the conventional way of tilling the soil in the area; deep loosening with a tine cultivator (DL) to a depth of 32 cm; and minimum tillage (MT) using a spring-tine cultivator to a depth of 0.10 m. The three agricultural treatments (MP, DL, MT) were laid out on the field following a Latin square design with three replicate experimental sub-plots per treatment. Each experimental sub-plot included a wheel track in about half of its width.

We performed the soil penetration resistance (PR) measurements on each experimental sub-plot with a fully automated penetrometer (30° angle cone with a base area of 1 cm^2^) mounted on a small vehicle as described in [2]. Such penetrometer measures soil strength at the intersection points of a 5 × 5 cm^2^ grid on the surface of an 80 × 80 cm^2^ area, and at different depths. It was developped to quantify the inner heterogeneity of soil volumes large enough to be representative of the modifications caused by tillage implements.

The measurements covered an area of 1.6 m (*x*-axis, perpendicular to the tillage direction) × 0.8 m (*y*-axis, in the tillage direction). The penetrometer was placed to cover equal areas of the soil affected and not affected by the machine traffic. Measurements were taken along a 5 cm resolution grid in the horizontal plane and between 0 and 50 cm depth with 1 cm increments, resulting in a 3D data array of 5, 5, and 1 cm resolution in the *x, y*, and *z*-direction, respectively. The measurements were conducted on bare soil at the beginning of the spring when the soil water content was expected to be near field capacity.

We averaged the 3D PR data array along the y-axis, resulting in a 2D vertical grid of 5 cm resolution in the *x*-axis and 1 cm resolution in the *z*-axis. We then performed linear interpolation to generate 1-cm resolution vertical PR data grids. We then carried out linear interpolation to create PR data grids with a 1 cm resolution on the and z-axis. The PR sensor threshold was 100 kg/cm^²^. Higher values were dismissed as they cannot be reliably interpreted.

The measurements and sampling were conducted on bare soil at the beginning of the spring, when the soil water content was expected to be near field capacity.

### 3.2 Soil core sampling and laboratory analysis

The soil core sampling campaign was conducted on the same field as the PR measurements but on two experimental sub-plots only: one with moldboard plowing (MP) and the other with minimum tillage (MT). We dug 1.6 m wide × 0.5 m deep pits perpendicularly to the soil tillage. An *in-situ* analysis was performed on these sections to identify and delineate the dominant soil structural units, such as clods and compacted zones, according to the methodology initially developed by [4]. We then sampled 13 undisturbed 100-cm³ soil cores in a sub-plot treated with MP and 14 samples in a sub-plot treated with MT. Sampling locations were selected to have an even surface coverage while representing the structural units identified with the soil profiling.

We measured the Bulk density (BD, in g.cm^−3^) of the soil samples using Eq. (1) where $W_{dry}$ is the soilsample's weight in grams after oven-drying for 48 h at 105 °C, and *V* is the volume of the soil sample in cm^3^ (here, 100 cm^3^).

(1) $$BD=\frac{W_{dry}}{V}$$

We measured the volumetric soil water content after reaching equilibrium at increasing levels of suction, calculated as the volume of water related to the volume of an oven-dried sample (soil core) [6] using the following equation:

(2) $$\theta_{v}\left(h\right)=\frac{\left(W_{h}-W_{dry}\right)/\rho_{w}}{V}$$

where $\theta_{v}\left(h\right)$ and $W_{h}\;$ are the soil water content (cm^3^.cm^−3^) and the weight (g) of the soil core at matrix potential *h* (cm)*,*$\;\rho_{w}$ is the water density set at 1 g.cm^−3^, and *V* is the soil core volume, here 100 cm^3^. We used a sandbox apparatus (® Eijkelkamp) for applying suction levels (*h*) equal to 1, 10, 31, and 63 cm (pF0, 1, 1.5, and 1.8 respectively), and low and high-pressure chambers (® Eijkelkamp) for suctions equal to 100, 310, and 1000 cm, and 3100, 10,000 and 13,100 cm, respectively (pF 2, 2.5, 3, 3.5, and 4.1, respectively).

Anomalous water retention curves were eliminated based on a visual inspection of the plotted experimental data points and the following criteria: (i) non-monotonic decrease in water content with increasing suction; or (ii) the water content at pF0 was higher than the porosity calculated using Eq. (3), where $\rho_{s}\;$ is the soil density estimated at 2.65 g/cm^3^.

(3) $$Por=1-BD/\rho_{s}$$

### 3.3 Parameters of the vanGenuchten's equation

The water retention curve for each sample was determined by fitting the vanGenuchten's equation (Eq. (4), [3])

(4) $$\theta_{v}\left(h\right)=\theta_{v,r}+\frac{\theta_{v,s}-\theta_{v,r}}{{\left(1+{\left(\propto \left|h\right|\right)}^{n}\right)}^{m}}$$

where h the soil matric head (cm), $\theta_{v}\left(h\right),\;\theta_{v,r}\;$ and $\theta_{v,s}$ the volumetric water content at matric head h, residual, and at saturation respectively (cm³.cm^−3^), and ∝ (cm^−1^), n (-), and m=1−(1/n)(-) are empirical curve-fitting parameters. Values of $\theta_{v,r}\;$ were constrained to 0. We used the *fminsearch* function in Matlab [7], a nonlinear programming solver that searches for the parameter combination that minimizes a user-defined objective function, defined as the root mean square error. To orient the search in the parameter space, initial values for $\theta_{v,s}$, ∝, and n parameters were set to 0.3, 0.5, and 1, respectively.

**2D grids of Bulk density**

The relationship between BD and PR established using the method detailed in (Alonso et al., In Press; it is the companion paper) was used to generate 2D grids of Bulk density from the PR 2D data grids. The relationship was applied until a 25 cm depth, after which the relationship was no longer reliable.

## Ethics Statement

The authors have read and followed the ethical requirements for publication in Data in Brief. Neither human study participants, animals, nor social media platforms were involved in collecting the data presented here.

## CRediT authorship contribution statement

**Alice Alonso:** Data curation, Formal analysis, Writing – original draft, Visualization. **Manuel Froidevaux:** Methodology. **Mathieu Javaux:** Conceptualization, Supervision, Writing – review & editing. **Eric Laloy:** Methodology. **Samuel Mattern:** Methodology. **Christian Roisin:** Conceptualization, Resources. **Marnik Vanclooster:** Conceptualization. **Charles Bielders:** Supervision, Conceptualization, Writing – review & editing, Project administration, Funding acquisition.

## Data Availability

Fine resolution profile-scale data to study the impact of treatment and machine traffic on agricultural soil structure and soil hydrologic properties (Original data) (Mendeley Data)
